# Investment literacy, social influence and undergraduates’ readiness to invest: dataset from Malaysia

**DOI:** 10.1016/j.dib.2020.106700

**Published:** 2020-12-26

**Authors:** Zairihan Abdul Halim, Muhammad Nukman Zolkefli, Suhal Kusairi, Safwan Mohd Nor, Nur Haiza Muhammad Zawawi, Muhammad Najit Sukemi

**Affiliations:** a Faculty of Business, Economics, and Social Development, Universiti Malaysia Terengganu, 21030 Kuala Nerus, Terengganu, Malaysia; b Victoria Institute of Strategic Economic Studies (VISES), Victoria University, City Flinders Campus, Melbourne, Victoria 3000, Australia

**Keywords:** Investment literacy, Stock investment, Demography, Readiness, Young adults

## Abstract

Since the launch of the InvestSmart™ initiative in 2014, the government agencies in Malaysia have been actively engaging community and university students via their outreach programs to promote investment literacy. Given this background, the state of the investment literacy of Malaysian undergraduates and their readiness to invest is intriguing. Therefore, this article offers a dataset of Malaysian undergraduates’ readiness to invest and the role that investment literacy and social influence play in their readiness to invest. Using a non-probability sampling technique, 500 undergraduate students in Malaysia were engaged to participate voluntarily in this survey. Descriptive statistics are presented in this paper. The dataset provides insights into the current state of investment literacy among Malaysian undergraduates, the sources of information on stock investment, and the readiness of these undergraduates to participate in the stock market.

**Specifications Table**

**Table:** 

Subject	Financial economics
Specific subject area	Financial literacy, stock investment
Type of data	Table
How data were acquired	Fieldwork and online survey
Data format	Raw, processed, descriptive
Parameters for data collection	We applied a non-probability sampling procedure, distributing both printed and online form questionnaires to student representatives at different universities, who were contacted before questionnaires were distributed.
Description of data collection	Our survey was conducted from December 2019 to April 2020. The questionnaires were distributed through fieldwork and an online form. In total, 500 undergraduate students participated voluntarily in the survey.
Data sources location	Higher Education Institutions in Malaysia
Data accessibility	With the article

## Value of the Data

• This dataset allows the analysis of the current state of investment literacy and readiness to participate in the stock market of a population of young adults in Malaysia (i.e. undergraduates in Malaysian universities).
• The dataset will inform the implementation of the National Strategy for Financial Literacy 2019–2023.
• Useful insights can be extracted from this dataset to assist finance or investment curriculum design at the university level.
• Researchers may employ this dataset to empirically examine the association between investment literacy, social influence, demographic factors and undergraduates’ readiness to invest.

## 1 Data Description

Since the official launch in 2014 of the nationwide investment-literacy campaign by Malaysia's capital market authority, the government agencies and professional bodies have been taking the initiative to engage the youth population, including university students, via their outreach programs. We provide a dataset describing investment literacy, sources of information and influence on stock investment, and readiness to participate in stock investment among Malaysian undergraduate students. Questionnaires were distributed online and through fieldwork. A total of 500 undergraduate students enrolled in private and public universities in Malaysia between December 2019 and April 2020 participated voluntarily in the survey.

The dataset has three major groups. Part A contains the demographic data of gender, race, area of study, parents’ combined income and college/university zone. Part B contains investment-literacy scores (gathered through 17 quiz-type questions in the questionnaire) that measure participants’ financial knowledge about stocks, bonds, interest rates, and mutual funds. All responses to the questions in this section were rated with a score of one for a correct response and a score of zero for an incorrect response. Part C contains data on social influence, i.e. the sources of information and influence of stock investment. Part D contains data of the investment readiness index. The questionnaire is provided as a supplementary file. Table 1 presents the frequency distribution of our respondents across demographic background, specifically gender, race, area of study, and parents’ combined income.

**Table 1:** Demographic information of respondents (n = 500).

Item		Freq (n)		Percentage (%)
				
Gender				
	Male	219		43.6
	Female	281		56.2
				
Race				
	Malay	334		66.8
	Chinese	106		21.2
	Indian	48		9.6
	Other	12		2.4
				
Area of study				
	Arts, humanities, and social sciences	136		27.2
	Business, economics, and management	198		39.6
	Engineering and technology	119		23.8
	Science and mathematics	40		8
	Other	7		1.4
				
Parents' combined monthly income				
	Less than RM2,000	144		28.8
	RM2,001–RM6,000	208		41.6
	RM6,001–RM10,000	86		17.2
	RM10,001 and above	62		12.4
				

Table 2 reports the frequency of participants’ responses to investment-related questions. More than 50% of respondents correctly answered several questions about mutual funds and the risk–reward relationship in stock investing. Participants’ knowledge of the function of the stock market and the effect of increasing interest rates on bonds is rather weak.

**Table 2:** Responses to investment-literacy questions (17 items).

	Item	Correct Answer Freq (n)	(%)	Incorrect Answer Freq (n)	(%)
1.	Investment in a stock provides share of ownership in a company.	173	34.6	327	65.4
2.	Investment in a bond provides a way for investors to lend money to the company.	211	42.2	289	57.8
3.	Every stock market performs the following function: It allows common stock to be traded.	195	39	305	61
4.	In general, the higher the risk involved in an investment, the greater the return that the market expects from this investment.	273	54.6	227	45.4
5.	Suppose you had RM100 in a savings account and the interest rate was 2% per year. After 5 years, how much do you think you would have in the account if you left money to grow? More than RM102	339	67.8	161	32.2
6.	If interest rates rise, what will typically happen to bond prices? They will fall	166	33.2	334	66.8
7.	If you hold shares of a company, then: whether you have long-term or short-term holdings, you are ashareholder of the company.	281	56.2	219	43.8
8.	Which of the following statements describes the main function of the stock market? The stock market brings people who want to buy stocks togetherwith those who want to sell stocks	218	43.6	282	56.4
9.	Normally, which asset has the highest fluctuations over time? Stocks	222	44.4	278	55.6
10.	When an investor spreads their money among different assets, the risk of losing money decreases.	238	47.6	262	52.4
11.	What happens if you buy a company's stock? You own a part of the company/You can vote on shareholderresolutions	264	52.8	236	47.2
12.	A stock mutual fund combines the money of many investors to buy a variety of stocks. True	379	75.8	121	24.2
13.	If you were to invest 1,000 in a stock mutual fund, it would be possible to have less than 1,000 when you withdraw your money. True	297	59.4	203	40.6
14.	An investment with a high return is likely to be a high-risk investment. True	366	73.2	134	26.8
15.	Mutual funds have a sure yield that depends on their previous yield. False	194	38.8	306	61.2
16.	In the long term, the value of stocks is more volatile than the value of bonds. True	293	58.6	207	41.4
17.	When the general level of interest rates increases, the value of bonds also increases. False	207	41.4	293	58.6
	Answered all 17 questions correctly	3	0.6		

Table 3 presents the participants’ responses about the sources of information and sources of influence of their stock investments. Our data indicate that friends and the internet are important sources of information and influence for stock investment.

**Table 3:** Responses to sources of influence of stock investment (7 items).

Item		Never (n) (%)	Rarely (n) (%)	Sometimes (n) (%)	Many times (n) (%)	Always (n) (%)
In relation to stock investment, how often you were influenced by, learned from, or discussed with the following:						
	Parents	101 (20.2)	79 (15.8)	152 (30.4)	124 (24.8)	44 (8.8)
	Friends	49 (9.8)	79 (15.8)	182 (36.4)	146 (29.2)	44 (8.8)
	Internet	15 (3.0)	41 (8.2)	141 (28.2)	180 (36.0)	123 (24.6)
	Public seminar or class	64 (12.8)	69 (13.8)	146 (29.2)	137 (27.4)	84 (16.8)
	Financial planner or advisor	94 (18.8)	75 (15.0)	137 (27.4)	102 (20.4)	92 (18.4)

Table 4 reports the mean and standard deviations for the investment readiness index across demographic factors. On average, the male respondents have a higher readiness index than the female respondents. Consistent with Khan et al. [3], across ethnicity, Chinese respondents have a higher degree of investment readiness than other ethnicities. Students enrolled in mathematics and science degrees have the highest average investment readiness index, followed by students enrolled in business, economics, and management degrees. In addition, students from middle to upper middle income backgrounds have a higher average investment readiness index compared to those of other income groups.

**Table 4:** Index of investment readiness across demographic background.

	Investment Adoption Readiness	
	Mean	Std Dev.
Gender		
Female	1.74	1.66
Male	2.25	1.72
		
Race		
Malay	1.84	1.70
Chinese	2.32	1.67
Indian	2.25	1.73
Other	0.92	1.44
		
Area of Study		
Arts, humanities, and social sciences	1.42	1.54
Business, economics,		
and management	2.24	1.78
Engineering and technology	1.82	1.57
Science and mathematics	2.75	1.75
Other	2.14	1.68
		
Parents’ combined monthly income		
Less than RM2,000	1.43	1.55
RM2,001–RM6,000	2.12	1.70
RM6,001–RM10,000	2.37	1.80
RM10,001 and above	2.06	1.67
		

## 2 Experimental Design, Materials and Methods

The primary data collection was performed through an online and field survey. Using a non-probability sampling technique (snowball sampling), a total of 500 printed questionnaires was initially distributed to identified contacts of selected universities located in the central, east coast, and northern regions of Malaysian peninsular. The response rate was 49.4% or 247 completed questionnaires. For the online survey, we created the questionnaire using the Google form. We contacted student representatives from universities outside regions which were not covered during field work. Using social media platform, we provided these students with a link to the online questionnaire and requested them to share the link to their fellow students at respective universities.

Our questionnaire design follows previous studies. Specifically, 17 quiz-type investment-literacy questions were adapted from Balloch et al. [1] and van Rooij et al. [6]. Items on sources of information about stock investment followed several studies, for example, Gerrans et al. [2] and Liang and Guo [4]. Our measure of readiness index was adapted from previous studies examining technology adoption readiness (e.g. [5]). To construct the investment readiness index, we measured participants’ readiness in terms of personal financial management, acquisition of knowledge and skill to invest in stocks, and their familiarity with trading platforms. An equally weighted index was then constructed from the participants’ responses on five yes/no questions capturing their readiness to invest in stocks, with 0 being ‘not ready at all’ and 5 being ‘most ready’.

## Ethics Statement

The questionnaire used for this research was approved by the Human Ethics Committee of Universiti Malaysia Terengganu. Informed consent was obtained from all respondents before they participated the survey. Participants were informed that the survey was anonymous.

## Declaration of Competing Interest

The authors declare that they have no known competing financial interests or personal relationships that could have appear to influence the work reported in this paper.

## Appendix Supplementary materials

Image, application 1

Image, application 2
