# Supplementary material for: Investment literacy, social influence and undergraduates’ readiness to invest: dataset from Malaysia
Source: Data Brief. 2020 Dec 26;34:106700. doi: 10.1016/j.dib.2020.106700 (PMC7804600; doi:10.1016/j.dib.2020.106700)
Supplement: Supplementary file 2 [file mmc2.docx]

**QUESTIONNAIRE ON INVESTMENT LITERACY AND UNDERGRADUATES’ READINESS TO ADOPT STOCK INVESTMENT**

Dear valued respondents,

This study is being conducted by Dr. Zairihan Abdul Halim and her research team under the financial support of Fundamental Research Grant Scheme, Ministry of Education. You are kindly requested to participate in this survey as a part of our research. The purpose of this survey is to gather information on undergraduates’ investment literacy, social influence, and readiness to consider stock/equity investment. Your participation in this survey is voluntary. This questionnaire has been approved by the Human Ethics Committee of Universiti Malaysia Terengganu. All survey responses will be recorded anonymously. Data from this survey will be used solely for academic purpose and confidentiality will be maintained. Your participation in this survey is very much appreciated.

Thank you.

**PART A: RESPONDENT’S BACKGROUND**

Please tick ( / ) in the appropriate boxes.

| 1. | Gender : |  | Male |  | Female |  |  |
| --- | --- | --- | --- | --- | --- | --- | --- |
|  |  |  |  |  |  |  |  |
| 2. | Race : |  | Malay |  | Chinese |  | Indian |
|  |  |  | Others: _____ |  |  |  |  |
| 3. | Area of Study : |  | Art, Humanities, and Social Sciences |  | Business, Economics, and Management |  | Engineering and Technology |
|  |  |  | Sciences and Mathematics |  | Others:_______ |  |  |
|  |  |  |  |  |  |  |  |
| 4. | Parents’ : |  | Less than RM2,000 |  | RM6,001-10,000 |  |  |
|  | Combined |  | RM2,001-6,000 |  | RM10,001 and above |  |  |
|  | Monthly income |  |  |  |  |  |  |
|  |  |  |  |  |  |  |  |

**PART B: INVESTMENT LITERACY**

**i) Please answer the following questions by circling the right answer.**

**1.** Investment in a stock _____________________

1. Provides share of ownership in a company
2. Provides a way for investors to lend money to the company
3. Provides return of original principal and generates periodic interest payments to the investors through its maturity

2. Investment in a bond____________________

1. Provides share of ownership in a company
2. Provides a way for investors to lend money to the company
3. Makes investor partially liable for the repayment of a company’s debt, which is outstanding

3. Every stock market performs the following function:

1. It determines the level of interest rates
2. It allows common stock to be traded
3. It allows loan be made
4. It channels funds from lenders-savers to borrowers-spenders

4. In general, higher the risk involved in an investment, the ___________________

1. greater the return that the market expects from this investment
2. lower is the return that market demands from this investment
3. risk and return of a stock are inversely related

5. Suppose you had RM100 in a savings account and the interest rate was 2% per year. After 5 years, how much do you think you would have in the account if you left money to grow?

1. More than RM102
2. Exactly RM102
3. Less than RM102

6. If interest rate rises, what will typically happen to bond prices?

1. They will rise
2. They will fall
3. They will stay the same
4. There is no relationship

7. If you hold the shares of a company, then:

1. whether you have long-terms or short-term holdings, you are lending money to the company
2. whether you have long-terms or short-term holdings, you are a shareholder of the company
3. when you have long-term holdings, you are a shareholder of a company, when you have short-term holding, then you are lending money to the company

8. Which of the following statements describes the main function of the stock market?

1. The stock market helps to predict stock earnings
2. The stock market results in an increase in the price of stocks
3. The stock market brings people who want to buy stocks together with those who want to sell stocks

9. Normally, which asset has the highest fluctuations over time?

1. Saving accounts
2. Bond
3. Stocks

10. When an investor spreads their money among different assets, the risk of losing money_________

1. increases
2. decreases
3. stays the same

11. What happens if you buy a company’s stock?

1. You own a part of the company
2. You have lent money to the company
3. You are liable for the company’s debt
4. The company will return your original investment to you with interest
5. You can vote on shareholder resolutions

12. A stock mutual fund combines the money of many investors to buy a variety of stocks.

1. True
2. False

13. If you were to invest 1,000 in a stock mutual fund, it would be possible to have less than 1,000 when you withdraw your money.

1. True
2. False

14. An investment with a high return is likely to be a high-risk investment.

1. True
2. False

15. Mutual funds have a sure yield that depends on their previous yield.

1. True
2. False

16. In the long term, the value of stocks is more volatile than the value of bonds.

1. True
2. False

17. When the general level of interest rates increases, the value of bonds also increases.

1. True
2. False

**PART C: SOCIAL INFLUENCE**

**Rate the following influences on a scale of 1-5 (1=never, 2 = rarely, 3 = sometimes, 4 =many times, 5 = always).**

**i.** **In relation to stock investment, how often you are influenced by, learn from, or discuss with the following**:

1-Parents

| 1 | 2 | 3 | 4 | 5 |
| --- | --- | --- | --- | --- |

2- Friends

| 1 | 2 | 3 | 4 | 5 |
| --- | --- | --- | --- | --- |

3-Internet

| 1 | 2 | 3 | 4 | 5 |
| --- | --- | --- | --- | --- |

4-Public seminar or class

| 1 | 2 | 3 | 4 | 5 |
| --- | --- | --- | --- | --- |

5-Financial planner or advisor

| 1 | 2 | 3 | 4 | 5 |
| --- | --- | --- | --- | --- |

**PART D: READINESS TO ADOPT STOCK INVESTMENT**

Please tick (/) the correct response

Y – Yes N – No

| No | Questions | Y | N |
| --- | --- | --- | --- |
| 1. | I can allocate money so I have enough each month for stock investment |  |  |
| 2. | I have taken class/training/course/seminar to prepare for my stock investment |  |  |
| 3. | I understand how to utilize a stock investment platform |  |  |
| 4. | I will seek consultation before I invest in a particular stock |  |  |
| 5. | I understand the pros and cons of using stock investment platform. |  |  |
